# Bilateral multifocal water-clear cell parathyroid adenoma: A case report with literature review

**DOI:** 10.1016/j.radcr.2026.09.050

**Published:** 2026-10-07

**Authors:** Abdulwahid M. Salih, Aras J. Qaradakhy, Ari M. Abdullah, Rawa M. Ali, Shko H. Hassan, Abdullah A. Qadir, Harun A. Ahmed, Berun A. Abdalla, Fahmi H. Kakamad

**Affiliations:** a Department of Head & Neck Surgery, Smart Health Tower, Madam Mitterrand Street, Sulaymaniyah, Iraq; b Department of Radiology, Smart Health Tower, Madam Mitterrand Street, Sulaymaniyah, Iraq; c Department of Radiology, Shorsh Teaching Hospital, Sulaymaniyah, Iraq; d Department of Pathology, Sulaymaniyah Teaching Hospital, Sulaymaniyah, Iraq; e Department of Pathology, Smart Health Tower, Madam Mitterrand Street, Sulaymaniyah, Iraq; f Department of Pathology, Hospital for Treatment of Victims of Chemical Weapons, Mawlawy Street, Halabja, Iraq; g Department of Oncology, Hiwa Cancer Hospital, Sulaymaniyah, Iraq; h Department of Oncology, Smart Health Tower, Madam Mitterrand Street, Sulaymaniyah, Iraq; i Zad Organization, Saeed Kaban Street, Judi Tower, Sulaymaniyah, Iraq; j Department of Scientific Affairs, Smart Health Tower, Madam Mitterrand Street, Sulaymaniyah, Iraq; k Kscien Organization for Scientific Research (Middle East Office), Hamid Street, Sulaymaniyah, Iraq; l College of Medicine, Department of Clinical Sciences, University of Sulaimani, Madam Mitterrand Street, Sulaymaniyah, Iraq

**Keywords:** Water-clear cell parathyroid adenoma, Primary hyperparathyroidism, Parathyroidectomy, Histopathology, Multifocal adenoma, Case report

## Abstract

Water-clear cell parathyroid adenoma (WCCPA) is an exceptionally rare subtype of parathyroid adenoma characterized by large, clear, glycogen-rich cells. A small number of cases have been reported in the literature. This report presents a rare case of bilateral multifocal WCCPA in a young female.

A 34-year-old female presented with generalized bone pain and fatigue. Laboratory evaluation revealed hypercalcemia (12.9 mg/dL) and markedly elevated PTH (488 pg/mL), consistent with primary hyperparathyroidism (PHPT). Neck ultrasonography and computed tomography identified bilateral inferior parathyroid lesions extending retrosternally. The patient underwent bilateral inferior parathyroidectomy, revealing four encapsulated nodules. Histopathological examination demonstrated water-clear chief cells arranged in lobules and sheets, confirming multifocal WCCPA. Postoperatively, PTH and serum calcium normalized.

Review of eight published WCCPA cases (2016–2025) revealed a mean age of 58.8 years (range 34–80) and a male predominance (62.5%). Most cases (87.5%) presented with symptomatic PHPT. Imaging with ultrasound and Tc-99m sestamibi was frequently inconclusive, with false-negative results in about 50% of cases. All patients underwent surgical excision, resulting in complete biochemical normalization (100%). No recurrences were reported during follow-up periods up to 50 months.

WCCPA is a rare but noteworthy cause of PHPT. Definitive diagnosis requires histopathological confirmation, and surgical management leads to excellent outcomes.

## Introduction

Water-clear cell parathyroid adenoma (WCCPA) is an exceptionally rare variant of parathyroid adenoma composed predominantly or exclusively of large clear cells packed with glycogen and numerous cytoplasmic vacuoles, which produce the distinctive “water-clear” appearance under the microscope [1]. As a benign parathyroid neoplasm, WCCPA falls within the spectrum of parathyroid adenomas but is distinguished by this cellular morphology, and accurate diagnosis requires careful differentiation from other parathyroid lesions, including chief cell adenoma (the most prevalent type), oncocytic adenoma, lipoadenoma, and particularly water-clear cell hyperplasia, the latter involving multiple enlarged glands rather than a solitary adenomatous mass [1].

WCCPA is among the rarest parathyroid adenoma subtypes, occurring far less frequently than the related condition of water-clear cell hyperplasia [2]. Its scarcity is reflected in institutional experience, with reported prevalence as low as 0.15% among all parathyroid lesions examined [3]. Fewer than 40 cases have been documented in the English literature to date [4]. The condition is most commonly diagnosed in middle-aged to elderly patients, with median ages at diagnosis of 56 and 53 years in the two largest pooled series [3,4]. Presentation before the age of 40 years is distinctly uncommon; among 37 pooled cases, only three patients were younger than 40, and a single previously reported patient was aged 34 years [4,5].

Clinically, WCCPA presents a diagnostic challenge, as no pathognomonic clinical or biochemical features reliably distinguish it from other causes of parathyroid hormone excess [6]. Reported cases are characterized by tumors that are considerably larger than conventional adenomas yet biochemically indolent, with serum calcium levels disproportionately modest relative to tumor bulk [4]. Radiologically, the entity is notable for reduced sensitivity of Tc-99m sestamibi scintigraphy, attributed to the low mitochondrial content of water-clear cells, while ultrasound performs comparatively well although the lesions are frequently isoechoic to thyroid parenchyma [3,4]. Consequently, the diagnosis is typically established only on histopathological examination of the surgical specimen following parathyroidectomy performed for primary hyperparathyroidism (PHPT), underscoring the importance of meticulous pathological evaluation in identifying this variant [6].

This case report aims to expand the limited literature on WCCPA by presenting a rare case, emphasizing its clinical presentation, histopathological features, and the diagnostic challenges. All referenced sources were critically assessed for reliability, and the case report was prepared in accordance with the CaReL guidelines [7,8].

## Case presentation

### Patient information

A 34-year-old female presented with generalized bone pain and fatigue. She had no relevant medical history, took no chronic medications, and was a non-smoker. There was no family history of endocrine neoplasia documented.

### Clinical findings

General examination was unremarkable. Neck examination revealed no palpable thyroid nodules or cervical lymphadenopathy. Vital signs were stable. BMI and blood pressure were within normal limits.

### Diagnostic assessment

Routine hematology tests including complete blood count were within normal limits. Biochemistry revealed hypercalcemia with total calcium 12.9 mg/dL (ref. 8.6–10.2 mg/dL) and ionized calcium 1.54 mmol/L (ref. 1.10–1.40 mmol/L). The parathyroid hormone (PTH) level was 488 pg/mL (ref. 15–65 pg/mL). The 25-hydroxy-vitamin D was 10.3 ng/mL (ref. 30–50 ng/mL). Renal function was normal (creatinine 0.73 mg/dL) (ref. 0.5–0.9 mg/dL), and urinalysis showed 2–3 RBC/HPF with ++ pus cells. Neck ultrasonography reported normal-sized thyroid lobes with homogeneous echotexture and two tiny TIRADS-4 thyroid nodules (right mid-third 4.5 × 3 × 3 mm; left mid-third 3 × 3 × 2 mm). Posterior to the lower poles there were hypoechoic, hypovascular lesions: on the right, a retrosternal/paraspinal mass partially visualized measuring approximately 30 × 21 × 22 mm; on the left, two retrosternal nodules inferior to the left lower pole, the largest 19 × 7.7 mm and mildly vascular. No significant pathological cervical lymphadenopathy was noted (Fig. 1). Fine-needle aspiration of the bilateral thyroid nodules showed benign follicular nodule (Bethesda II). Computed tomography (CT) of the neck and chest was performed as a non-contrast (native) acquisition followed by a post-contrast phase. A dedicated four-dimensional (4D) parathyroid protocol (pre-contrast, arterial, and delayed phases) was not undertaken because the diagnosis of primary hyperparathyroidism had already been established on clinical, biochemical, and ultrasonographic grounds; CT was obtained primarily for anatomical localization of the partially retrosternal lesions and to exclude additional pathology not seen on ultrasound. For reference, a typical parathyroid lesion on multiphase CT shows low attenuation before contrast, brisk enhancement in the early arterial phase, and washout on the delayed phase. The study demonstrated enlarged posterior parathyroid glands: right posterior 4.0 × 2.3 × 2.0 cm adjacent to the esophagus (heterogeneous enhancement), left posterior 4.5 × 1.8 × 0.8 cm adjacent to the esophagus, plus smaller anterior nodules (right anterior 2.0 × 0.5 cm; left anterior 2.0 × 0.6 cm). Two posterior lesions extended inferiorly toward the mediastinum. All identified parathyroid lesions showed enhancement following contrast administration. The radiology impression was in favor of parathyroid hyperplasia/adenomas, with the right posterior lesion considered suspicious on the basis of heterogeneity, and recommended tissue diagnosis (Fig. 2). Tc-99m sestamibi scintigraphy was not performed, as the diagnosis had already been established biochemically and the lesions had been localized by ultrasound and CT; cross-sectional imaging was preferred given the partially retrosternal location of the lesions and the need to exclude other pathology, and scintigraphy was therefore not considered necessary for surgical planning.

**Fig. 1:**
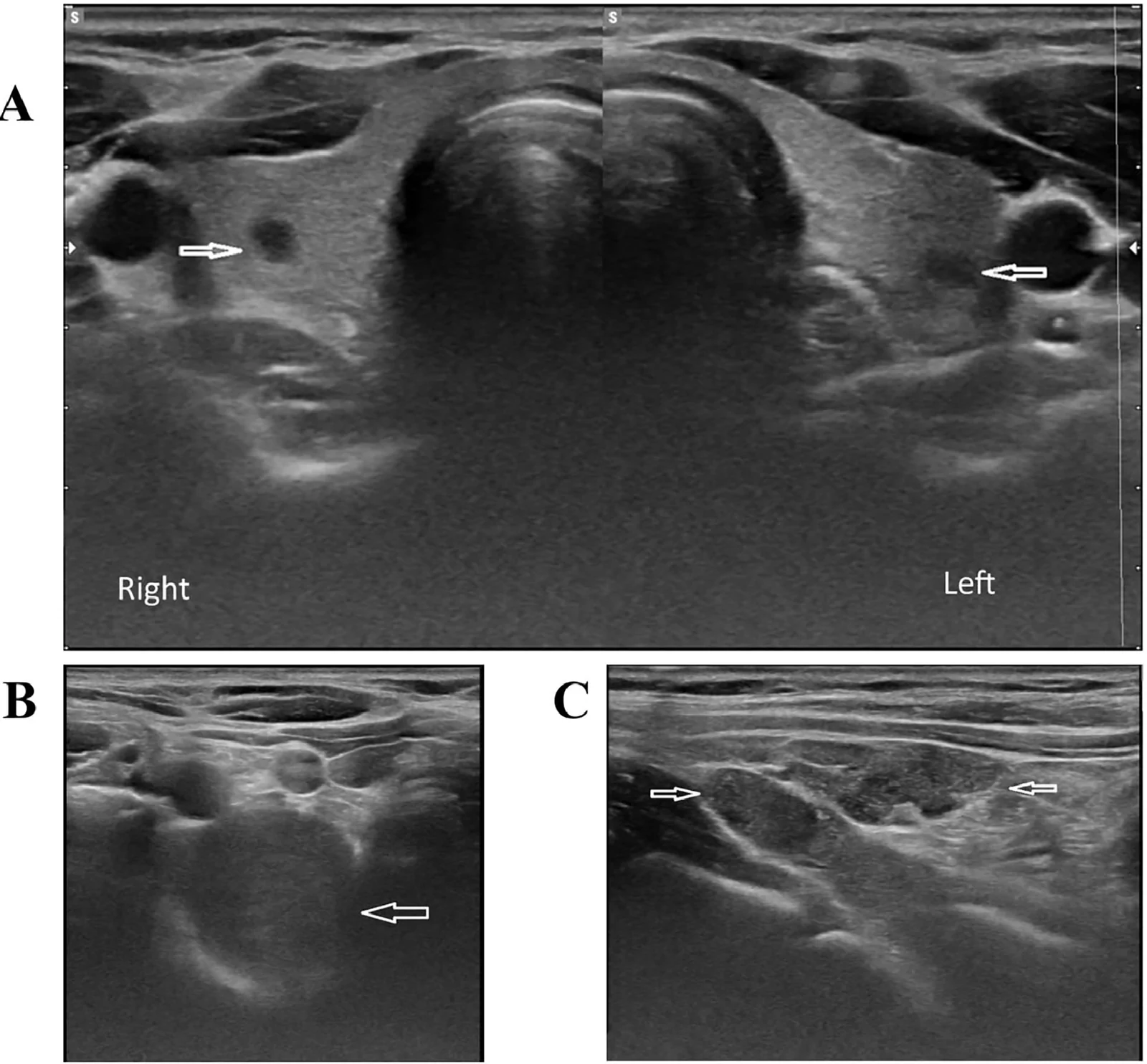
(A) Transverse section of thyroid gland revealing bilateral small solid hypoechoic nodules classified as TR4. (B) Right paraspinal solid hypoechoic lesion measuring 30 × 21 × 22 mm, suggestive of parathyroid pathology. (C) Left posterior-inferior aspect of the thyroid demonstrating two solid hypoechoic nodules with partial retrosternal location.

**Fig. 2:**
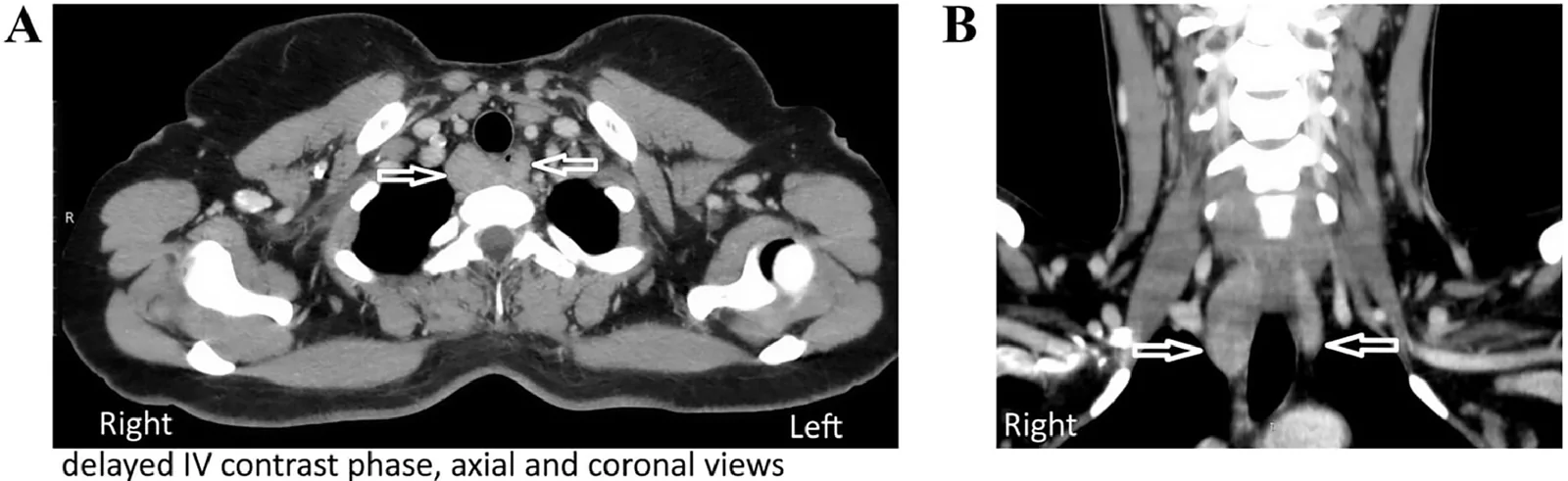
(A) Axial contrast-enhanced CT scan showing solid enhancing nodules on both the right and left sides of the esophagus. (B) Coronal contrast-enhanced CT scan demonstrating the same nodules described above.

### Therapeutic intervention

The patient underwent bilateral inferior parathyroidectomy under general anesthesia. The patient was positioned supine with the neck extended, and a standard low cervical incision was performed. Intraoperatively, bilateral enlargement of the inferior parathyroid glands was observed, with a right-sided retrosternal extension (Fig. 3). The excised tissues were sent for histopathological evaluation.

**Fig. 3:**
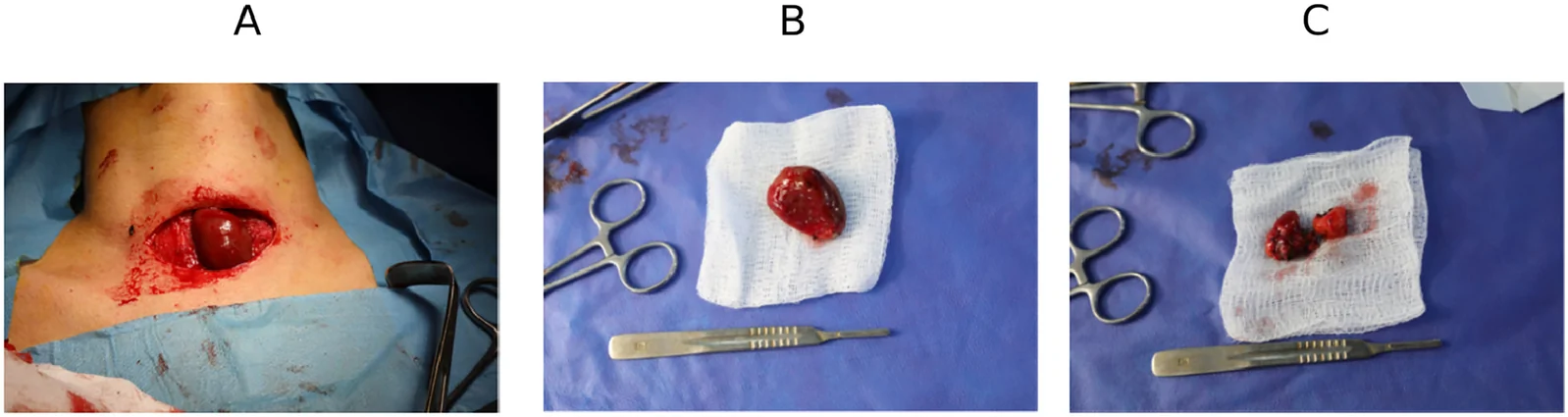
(A) Intraoperative exposure of the neck demonstrating a markedly enlarged right inferior parathyroid gland with retrosternal extension visualized through a standard low cervical (Kocher) incision. (B) Gross specimen of the right inferior parathyroidectomy showing an ovoid, well-circumscribed, brown, rubbery lesion measuring 4.2 × 3.0 × 2.0 cm. (C) Gross specimen of the left inferior parathyroidectomy comprising multiple nodules, the largest measuring 4.5 cm, consistent with multifocal water-clear cell parathyroid adenomas.

Microscopic examination demonstrated multiple encapsulated, well-circumscribed nodules composed predominantly of water-clear chief cells, arranged in lobular and sheet-like patterns separated by a delicate capillary network. The cells exhibited abundant, finely vacuolated, optically clear cytoplasm and small round nuclei with finely stippled chromatin. No capsular or vascular invasion, no cellular atypia, and zero mitotic figures per 50 HPF were identified. Regional lymph nodes were free of pathological involvement. The final diagnosis was multifocal WCCPA (Fig. 4). Involving four tumors: right (4.2 cm and 2.5 cm) and left (4.5 cm and 2.5 cm).

**Fig. 4:**
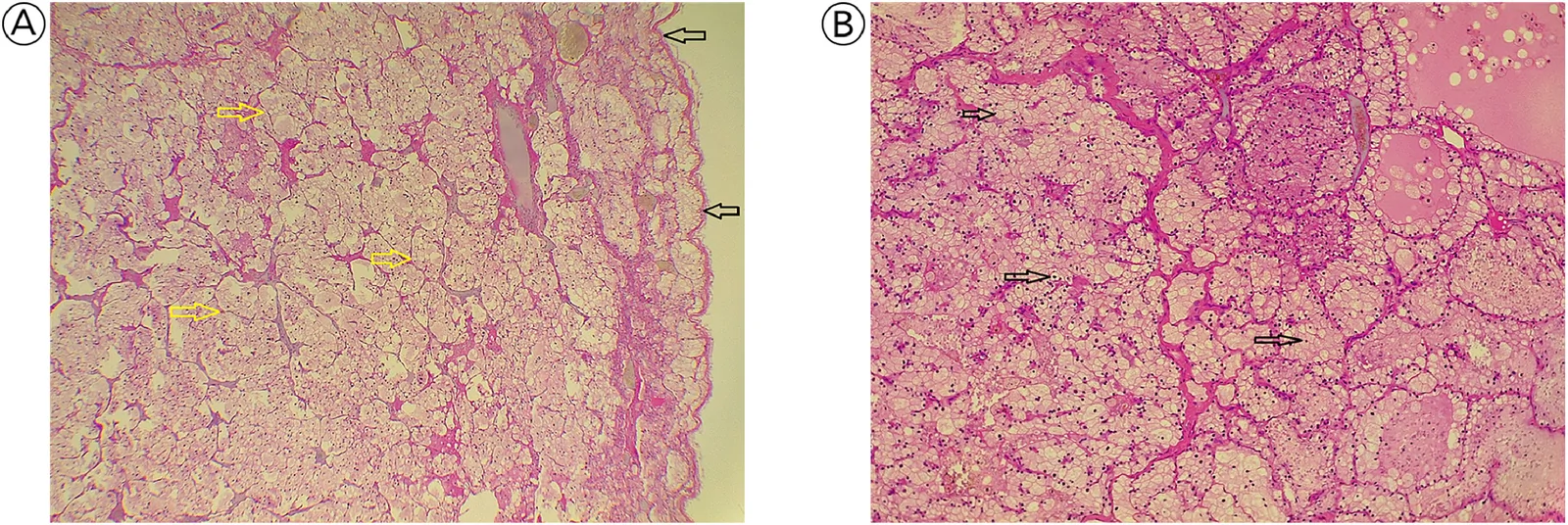
(A) Section reveals a well-defined mass with thin fibrous capsule (dark arrows), that composed of solid and trabeculae nests of uniform cells with clear cytoplasm (yellow arrows). Hematoxylin and Eosin stain, 4X. (B) Higher power showing large cells with small round nuclei, and abundant clear cytoplasm (dark arrows). Hematoxylin and Eosin stain, 10X.

### Follow-up and outcome

The early postoperative course was uneventful, and the patient was discharged on the first postoperative day. At follow-up, serum PTH had decreased to 57.1 pg/mL (reference range: 15–65 pg/mL) and serum calcium 9.5 mg/dL.

## Discussion

WCCPA typically affects older adults, most commonly in the sixth to seventh decade of life, with reported median ages at diagnosis of 56 and 53 years in the two largest pooled series [3,4]. Among the eight cases reviewed here a male predominance was observed [2,4,5,9,10,[11], [12], [13]]. The present case involved a 34-year-old female, representing one of the youngest documented patients to date; only two younger patients have been described, aged 18 and 33 years [4]. This notable age difference suggests that WCCPA, though traditionally regarded as a disease of older individuals, can also occur in younger adults, thereby broadening its recognized demographic profile.

Eight cases of WCCPA reported between 2016 and 2025 showed a mean age of 58.8 years (range, 34–80) with a male predominance (62.5%), contrasting with the slight female bias seen in conventional adenomas. Most patients (87.5%) presented with symptomatic PHPT, featuring hypercalcemia, bone pain, fatigue, or nephrolithiasis, while few were asymptomatic. Mean serum calcium were 10 mg/dL, and PTH levels commonly exceeded 400 pg/mL. Ultrasound and Tc-99m sestamibi scans were used in all cases, but about half showed false-negative results due to the low mitochondrial content of clear cells. Surgical excision led to complete biochemical normalization (100%), and histology consistently revealed clear, vacuolated cells arranged in lobules or sheets without atypia or invasion. All patients had favorable outcomes, with no recurrences during follow-up of up to 50 months. Overall, WCCPA remains a rare, benign, and curable parathyroid lesion, notable for its diagnostic challenges and excellent surgical prognosis [2,4,5,9,10,[11], [12], [13]] (Table 1).

**Table 1:** Summary of reported cases of water-clear cell parathyroid adenoma.

Study	Country	Age	Sex	PMH/PSH	Presentation	Lab	Imaging findings	Management	HPE	Post-op result/Outcome
Abdullah et al. [2]	Iraq	80	F	None reported	Fatigue, muscle pain	Ca 12.73 mg/dL ↑; PTH 303 pg/mL ↑	US 28 × 16 × 11 mm nodule posterior to left lobe with retrosternal extension	Left lower parathyroidectomy	Multifocal WCCPA (clear cells, no invasion)	Ca 9.31 mg/dL; PTH 42.4 pg/mL; uneventful
Gossili et al. [13]	Denmark	57	M	HTN, mild renal dysfunction	Asymptomatic severe hypercalcemia	Ca 3.11 mmol/L (12.4 mg/dL); PTH 303 pg/mL ↑	US 4.3 × 2.7 cm; MIBI +; ^11C-Met-PET +	Parathyroidectomy	Encapsulated WCCPA; chromogranin A +, no invasion	PTH ↓ > 50% intra-op; normalized Ca; uneventful
Durant et al. [10]	USA	70	M	Osteoporosis, HTN, Barrett’s esophagus	Small-bowel obstruction; hypercalcemia symptoms	Ca 12.7 mg/dL ↑; PTH 110.4 pg/mL ↑	US 2.7 × 2.1 cm mass; MIBI persistent uptake	Left superior parathyroidectomy	WCCPA (3.2 × 1.5 × 0.6 cm); no atypia or mitoses	Normal Ca 9.7 mg/dL; PTH 11.4 pg/mL; no recurrence
Yang et al. [4]	Taiwan	56	M	HTN, tophaceous gout	Bone pain, fatigue, nephrolithiasis	Ca 12.7 mg/dL ↑; PTH 937 pg/mL ↑; Ph 2.1 mg/dL ↓	US bilateral tumors; MIBI bilateral uptake	Bilateral inferior parathyroidectomy	Double WCCPA (5.6 & 2.7 cm); clear cells	Normocalcemia; PTH 15.5 pg/mL; uneventful
Mohamed et al. [11]	Qatar	56	F	Hypertension	Asymptomatic hypercalcemia; neck mass	Ca 3.13 mmol/L (12.5 mg/dL); PTH 1036 pg/mL	US 21 × 18 × 36 mm adenoma; MIBI +	Hemithyroidectomy + adenoma excision	Encapsulated WCCPA (3 × 2.5 × 1.5 cm; 9 g)	Normocalcemia; transient hypocalcemia
Arık et al. [9]	Turkey	70	M	Mild osteoporosis	Back pain; mediastinal mass	None reported	CT 6 cm mediastinal mass; PET-CT no uptake	Mediastinal mass resection	Ectopic WCCPA (6 × 2.5 × 2 cm); PTH +, CK +	Normocalcemia; PTH 63 pg/mL; 50-mo f/u
Yazar et al. [12]	Turkey	47	M	Recurrent nephrolithiasis	Abdominal pain, nausea, vomiting, lethargy; diagnosed with Hypercalcemic crisis	Ca 16.6 mg/dL ↑; PTH 744 pg/mL ↑	US bilateral lesions; MIBI –	Bilateral inferior parathyroidectomy	Double WCCPA (2.5 & 1.6 cm)	Normocalcemia & normal PTH 48 mo
Pirela et al. [5]	USA	34	F	Family history: thyroid cancer, Graves’ disease	Incidental goiter	Ca 9.3 mg/dL (normal); PTH not measured	US 3.6 × 2.6 × 3.3 cm right-lobe nodule	Right lobectomy + isthmusectomy	Intrathyroidal WCCA (2.5 × 1.7 × 1.5 cm); GATA3 +, PTH weak +	Transient hypocalcemia; benign course
Current case	Iraq	34	F	None	Generalized bone pain, fatigue	Ca 12.9 mg/dL ↑; ionized Ca 1.54 mmol/L ↑; PTH 488 pg/mL ↑; 25-OH vit D 10.3 ng/mL ↓	US bilateral hypoechoic retrosternal lesions (right 30 × 21 × 22 mm; left 19 × 7.7 mm); CT enlarged enhancing posterior parathyroid glands (right 4.0 × 2.3 × 2.0 cm; left 4.5 × 1.8 × 0.8 cm) with mediastinal extension; MIBI not performed	Bilateral inferior parathyroidectomy	Multifocal WCCPA; four encapsulated nodules (right 4.2 & 2.5 cm; left 4.5 & 2.5 cm); clear cells, no atypia or invasion	Ca 9.5 mg/dL; PTH 57.1 pg/mL; uneventful, discharged POD1

Abbreviations: -, negative; +, positive; Ca, calcium; CT, computed tomography; F, female; HPE, histopathological examination; M, male; MIBI, sestamibi scan; PMH, past medical history; PSH, past surgical history; PTH, parathyroid hormone; US, ultrasound; WCCPA, water-clear cell parathyroid adenoma.

Most previously reported cases of WCCPA presented with symptomatic PHPT, often manifesting as hypercalcemia, bone pain, or nephrolithiasis [13]. In the present case, the patient exhibited generalized bone pain and fatigue, accompanied by marked hypercalcemia (12.9 mg/dL) and elevated parathyroid hormone levels (488 pg/mL). By contrast, other reports, including those by Mohamed et al. and Pirela et al., described patients in whom the lesion was identified incidentally or during workup for unrelated thyroid pathology [5,11]. The presence of nephrocalcinosis in this case further underscores the systemic impact of chronic, untreated hyperparathyroidism.

Radiological evaluation in WCCPA serves to confirm an abnormal parathyroid gland and define its position for surgical planning, but the performance of the standard modalities differs from that seen in conventional PHPT, where ultrasound and Tc-99m sestamibi scintigraphy perform broadly comparably [4]. In the largest pooled analysis, ultrasound localized 89% of adenomas while scintigraphy was positive in only 60% [4], and an institutional series found scintigraphy inconclusive or negative in 58% of cases compared with 19% for ultrasound [3]. The explanation is cytological: sestamibi is retained within mitochondria, and the glycogen-rich, heavily vacuolated water-clear cells contain few mitochondria and few admixed oxyphil cells, producing rapid washout and a falsely negative scan despite substantial tumor size [3,4]. Ultrasound performs better because the ballooned, water-rich cytoplasm yields a large, conspicuous mass [3], although these lesions are often isoechoic to thyroid parenchyma and may be mistaken for thyroid nodules [4,10]. Ultrasound combined with cross-sectional imaging is therefore the more dependable strategy, and CT becomes essential for retrosternal or ectopic lesions, which have been reported in mediastinal, retro-esophageal and intrathyroidal locations [5,9]. No imaging finding is pathognomonic, and WCCPA cannot be diagnosed preoperatively on radiological grounds alone; several features should nonetheless raise suspicion. The most consistent is size, with a median maximum diameter of 3.8 cm and median weight of 5.27 g against 1–3 cm and 0.3–5 g for classic adenomas [4]. The most useful in practice is discordance between a large, well-defined lesion on ultrasound and a negative or equivocal sestamibi study, a combination distinctly unusual for a conventional adenoma of comparable bulk [3,4]. Loss of the expected proportionality between serum calcium and tumor burden provides a further clue, as a bulky gland with only modest hypercalcemia should prompt consideration of this variant [4]. In the present case, ultrasound and CT identified bilateral lesions with partial retrosternal extension, the largest measuring 4.5 cm, consistent with the large size characteristic of this entity, although the lesions were hypoechoic rather than isoechoic and the biochemical derangement was marked rather than indolent. The multifocal bilateral distribution also contrasts with the solitary lesions predominating in reported series, though all four previously reported double WCCPAs likewise showed a crossed bilateral distribution [4].

Histopathological examination remains the definitive diagnostic method, since WCCPA cannot be distinguished from a conventional adenoma on clinical or radiological grounds. The present case showed the expected appearance of encapsulated nodules composed of large, vacuolated clear cells without atypia, mitotic activity or capsular invasion [2,4]. Two aspects warrant comment. First, four separate tumors, two on each side, confirmed multifocal disease, a finding seldom reported and occasionally interpreted as hyperplasia rather than adenoma; a rim of compressed normal parathyroid tissue, when present, favors adenoma over water-clear cell hyperplasia [3], and several authors have proposed that these lesions lie on a morphological continuum rather than forming discrete entities [2,4]. Second, the patient's young age raised the possibility of MEN type 1, although genetic confirmation was not available.

Surgical resection remains the definitive treatment and is typically curative. In prior reports, parathyroidectomy led to normalization of serum calcium and PTH levels in nearly all patients [10,13]. Consistent with these findings, this patient’s postoperative PTH decreased markedly from 488 pg/mL to 57 pg/mL. This biochemical profile may reflect residual hyperfunctioning tissue, “hungry-bone” syndrome, or delayed calcium homeostasis, phenomena only briefly mentioned in previous studies. Such persistence emphasizes the need for long-term follow-up, especially when multifocal disease is suspected. Comparison with prior reports reveals both diagnostic and conceptual controversies surrounding WCCPA. While earlier authors have characterized WCCPA as a benign and typically solitary lesion [2,9,10], the present case demonstrates that it may also occur bilaterally and multifocally, even in younger adults. These findings raise important questions regarding its possible association with parathyroid hyperplasia or familial endocrine syndromes, particularly MEN type 1. Further molecular, immunohistochemical, and genetic investigations are warranted to clarify whether multifocal WCCPA represents multiple adenomas or a hyperplastic variant within the same biological continuum [4,9]. In our case, however, molecular and genetic testing for MEN1 and related mutations could not be performed due to the unavailability of such analyses in Iraq.

This case has several limitations that should be acknowledged. First, molecular and genetic analyses for MEN1 and related mutations could not be performed in our case due to the unavailability of such testing facilities in Iraq, which limited further confirmation of potential hereditary or molecular associations. Second, intraoperative PTH monitoring was not conducted, preventing real-time biochemical verification of complete adenoma excision. Additionally, the diagnostic imaging was confined to ultrasound and CT, without functional studies such as Tc-99m sestamibi or PET/CT, which might have provided more precise localization and comparative data. Finally, the postoperative follow-up period was relatively short, making it difficult to assess long-term calcium and PTH stability or potential recurrence.

## Conclusion

Water-clear cell parathyroid adenoma is a rare but curable cause of PHPT that may present bilaterally and multifocally, including in young adults. No clinical or radiological feature is pathognomonic, though a large lesion clearly seen on ultrasound yet negative on sestamibi scintigraphy should raise suspicion. Definitive diagnosis requires histopathological confirmation, and complete surgical excision leads to excellent outcomes.

## Ethical approval

This retrospective case report was conducted in accordance with the ethical principles of the Declaration of Helsinki and its subsequent amendments.

## Patient consent

Written informed consent was obtained from the patient for publication of the clinical details and accompanying images. All identifying information has been removed to protect patient confidentiality and privacy.
